# Building foundations: Invited commentary: The clinicopathological landscape of thyroid cancer in South Africa: A multi‐institutional review

**DOI:** 10.1002/wjs.12392

**Published:** 2024-10-29

**Authors:** Kristin Long

**Affiliations:** ^1^ University of Wisconsin School of Medicine and Public Health ‐ Surgery Madison Wisconsin USA

With increasing efforts on optimizing management of thyroid cancer diagnoses, understanding regional disease variability and regionally specific resource challenges will be vital. The manuscript entitled “The Clinicopathological Landscape of Thyroid Cancer in South Africa” by Dr. Conradie and colleagues presents an important comprehensive review of thyroid cancer patients in a unique social, economic, & geographical region and will serve as a foundation for developing national quality registries and context‐appropriate guidelines for care.^1^

The paper provides important data on thyroid cancer statistics in a developing country with wide variability in resource levels, where treatment often remains suboptimal.^2^ The authors are particularly to be commended for the collaborative multi‐institutional work, representing five of the nine provinces in the country. As any researcher who has worked on large scale efforts can attest, following complex projects such as these through to fruition is a labor of love. Competing demands for time allow less flexibility for academic endeavors in strained clinical environments, as seen by the number of institutions who were initially recruited but ultimately unable to complete the data collection. Additionally, the authors note that no private health care sector cases were included in the study, highlighting a common difficulty of accurately representing a population with varying access to both public and private resources. Finally, as is extremely common in lower resourced settings, data collected is often incomplete, unverified, and limited in scope, with little to no support for this work at an institutional or health‐system level. While this struggle is not unique to South Africa, it does clearly highlight that more resource‐depleted clinical environments have even less bandwidth to contribute to important studies such as this, and the data presented provides a strong argument as to why all hospital systems and surgeons alike should prioritize clinical research and quality improvement.

Surgical outcomes can improve with surgeon volume and experience, and collecting both patient/disease specific characteristics as well as surgeon technique, resource use, and outcomes will support a truly comprehensive national registry than can further regional improvement efforts.^3^ Interventions cannot be successfully or optimally implemented without a solid characterization of the disease landscape, which differs widely across the globe. For example, this paper highlights that despite widespread iodination of table salt for more than 40 years, the incidence of follicular thyroid carcinoma remains high in South Africa, suggesting more disease complexity than simple iodine deficiency.

Protocols popular in higher income countries may not work well in lower resource settings, and as the authors note, are not universally applicable to areas as diverse as South Africa. Identifying current practice patterns helps to highlight resource struggles and can facilitate support for better data collection, outcomes monitoring, and overall quality improvement. A critical point is made in this paper, noting that less than 20% of preoperative ultrasounds were reported using TIRADS criteria, which is well established in other countries and represents a key point of process improvement. Ultrasound is the most routinely available imaging modality in many of the lowest resource settings, and expertise with this technique should be prioritized.^4^

Not surprisingly, an overwhelming majority of the patients in this series presented with a palpable mass, leading to more advanced disease on final pathology and presenting a marked contrast to higher income countries. Longer term outcomes will be interesting to follow in this population and elucidate more about the natural history of the disease, as even though the tumors were larger in size or had more aggressive features, they remain well‐differentiated thyroid cancers with likely very good prognosis.

As the authors conclude, this first national review of thyroid cancer in South Africa provides valuable insight into regionally unique disease experiences and surgical challenges. From the high proportion of clinically evident tumors at diagnosis to the low rates of formal ultrasound classification and limitations of cytology and other resources, accurate characterization of thyroid cancer and its treatment in developing regions will be the cornerstone of public health initiatives designed to improve surgical care and capacity worldwide.

## AUTHOR CONTRIBUTIONS

**Kristin Long**: Conceptualization; writing – original draft; writing – review & editing.

## CONFLICT OF INTEREST STATEMENT

The author declare no conflicts of interest.

## ETHICS STATEMENT

Not applicable.
